# Mast Cells Density Positive to Tryptase Correlates with Angiogenesis in Pancreatic Ductal Adenocarcinoma Patients Having Undergone Surgery

**DOI:** 10.1155/2014/951957

**Published:** 2014-06-04

**Authors:** Michele Ammendola, Rosario Sacco, Giuseppe Sammarco, Giuseppe Donato, Valeria Zuccalà, Maria Luposella, Rosa Patruno, Ilaria Marech, Severino Montemurro, Nicola Zizzo, Cosmo Damiano Gadaleta, Girolamo Ranieri

**Affiliations:** ^1^Department of Medical and Surgery Sciences, Clinical Surgery Unit, University of Catanzaro “Magna Graecia” Medical School, Viale Europa, Germaneto, 88100 Catanzaro, Italy; ^2^Surgery Unit, National Cancer Research Centre, Giovanni Paolo II, 70100 Bari, Italy; ^3^Health Science Department, Pathology Unit, University of Catanzaro “Magna Graecia” Medical School, 88100 Catanzaro, Italy; ^4^Department of Medical and Surgery Sciences, Cardiovascular Disease Unit, University of Catanzaro “Magna Graecia” Medical School, 88100 Catanzaro, Italy; ^5^Interventional Radiology Unit with Integrated Section of Translational Medical Oncology, National Cancer Research Centre, Giovanni Paolo II, 70100 Bari, Italy; ^6^Chair of Pathology, “Aldo Moro” University of Bari, 70100 Bari, Italy

## Abstract

*Background*. Literature data suggest that cells such as mast cells (MCs), are involved in angiogenesis. MCs can stimulate angiogenesis by releasing of several proangiogenic cytokines stored in their cytoplasm. In particular MCs can release tryptase, a potent *in vivo* and *in vitro* proangiogenic factor. Nevertheless few data are available concerning the role of MCs positive to tryptase in primary pancreatic cancer angiogenesis. This study analyzed MCs and angiogenesis in primary tumour tissue from patients affected by pancreatic ductal adenocarcinoma (PDAC). *Method*. A series of 31 PDAC patients with stage T_2-3_N_0-1_M_0_ (by AJCC for Pancreas Cancer Staging 7th Edition) was selected and then underwent surgery. Tumour tissue samples were evaluated by means of immunohistochemistry and image analysis methods in terms of number of MCs positive to tryptase (MCDPT), area occupied by MCs positive to tryptase (MCAPT), microvascular density (MVD), and endothelial area (EA). The above parameters were related to each other and to the main clinicopathological features. *Results*. A significant correlation between MCDPT, MCAPT, MVD, and EA group was found by Pearson's *t*-test analysis (*r* ranged from 0.69 to 0.81; *P* value ranged from 0.001 to 0.003). No other significant correlation was found. *Conclusion*. Our pilot data suggest that MCs positive to tryptase may play a role in PDAC angiogenesis and they could be further evaluated as a novel tumour biomarker and as a target of antiangiogenic therapy.

## 1. Introduction

Inflammatory cells, such as macrophages, lymphocytes, and mast cells (MCs), play a major role in tumour angiogenesis by means of angiogenic cytokines stored in their cytoplasm. MCs are involved in neovascularization in experimentally induced tumour, accumulate near to tumour cells before the angiogenesis onset, and participate in the metastatic spreading of primary tumours. MCs intervene in angiogenic process releasing classical proangiogenic factors, such as vascular endothelial growth factor (VEGF), thymidine phosphorylase (TP), fibroblast growth factor-2 (FGF-2), and the nonclassical proangiogenic factor, namely, tryptase stored in their secretory granules [1–9]. The role of MCs has been broadly studied in benign lesions, in animal and human's cancers, such as keloids, mast cells tumours, and head and neck, colorectal, gastric, lung, and cutaneous malignancies, indicating that MCs density is highly correlated with the extent of tumour angiogenesis [10–14]. Recent data have shown that MCs density is correlated with angiogenesis and progression of patients with pancreatic cancer [15, 16]. However, no data have been published regarding the correlation each to other of MCs density positive to tryptase (MCDPT), area occupied by MCs positive to tryptase (MCAPT), microvascular density (MVD), endothelial area (EA) and the main clinicopathological features in primary tumour tissue of affected patients. To this end, we conducted a prospective study in a series of 31 pancreatic ductal adenocarcinoma patients (PDACP) having undergone surgery with stage T_2-3_N_0-1_M_0_ (by AJCC for Pancreas Cancer Staging 7th Edition). Tumour tissue samples were evaluated by means of immunohistochemistry and image analysis methods, obtaining a significant correlation between MCDPT, MCAPT, MVD, and EA group. Our pilot data suggest that MCs positive to tryptase may play a role in PDAC angiogenesis and they could be further evaluated as a novel tumour biomarker and as a target of antiangiogenic therapy.

## 2. Patients and Methods 

### 2.1. Patients

The clinicopathological features of selected patients are summarized in Table 1. A total of 31 PDACP patients underwent potential curative resection. Surgical approaches used were pancreaticoduodenectomy, distal pancreatectomy, and total pancreatectomy with lymph node dissection. Patients were staged according to the American Joint Committee on Cancer 7th Edition (AJCC-TNM) classification and the World Health Organization classification (2000 version) was used for pathologic grading. All patients had no distant metastases on computed tomography and ten patients had received neoadjuvant-therapy based on Gemcitabine or FOLFIRINOX. The study was approved by the Ethics Committee of “Mater Domini” Hospital, “Magna Graecia” University, Catanzaro, and from each enrolled patient the signed informed consent was obtained.

### 2.2. Immunohistochemistry

For the evaluation of MCDPT, MCAPT, MVD, and EA, a three-layer biotin-avidin-peroxidase system was utilized [17]. Briefly, 4 *μ*m thick serial sections of formalin-fixed and paraffin-embedded surgically removed tumour samples were deparaffinised. Then, for antigen retrieval, sections were microwaved at 500 W for 10 min, after which endogenous peroxidase activity was blocked with 3% hydrogen peroxide solution. Next, adjacent slides were incubated with the monoclonal antibodies anti-CD31 (clone JC70a; Dako) diluted 1 : 40 for 30 min at room temperature and anti-tryptase (clone AA1; Dako, Glostrup, Denmark) diluted 1 : 100 for 1 h at room temperature. The bound antibody was visualised using biotinylated secondary antibody, avidin-biotin peroxidase complex, and fast red. Nuclear counterstaining was performed with Gill's haematoxylin number 2 (Polysciences, Warrington, PA, USA). Primary antibody was omitted in negative controls.

### 2.3. Morphometric Assay

An image analysis system (Semiquantimet 400 Nikon) was employed.

**Figure 1:**
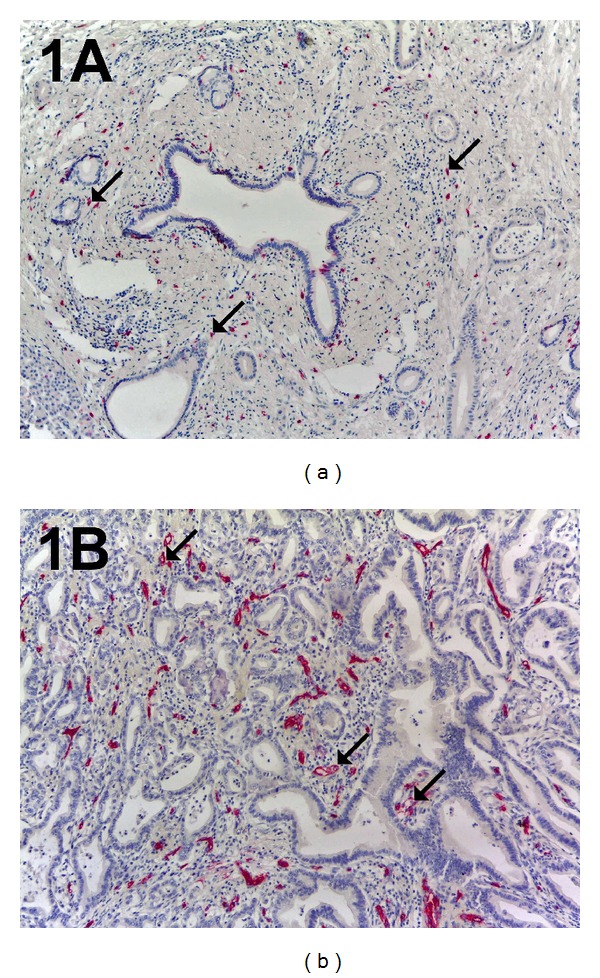
In (a) a pancreatic ductal adenocarcinoma sample stained with the anti-tryptase antibody. Many scattered red immunostained MCs. Arrows indicate single MC. Magnification: in (b) a highly vascularized pancreatic ductal adenocarcinoma sample stained with the anti-CD-31 antibody. Many red immunostained microvessels. Arrows indicate microvessel. Magnification: (a-b), ×100.

**Figure 2:**
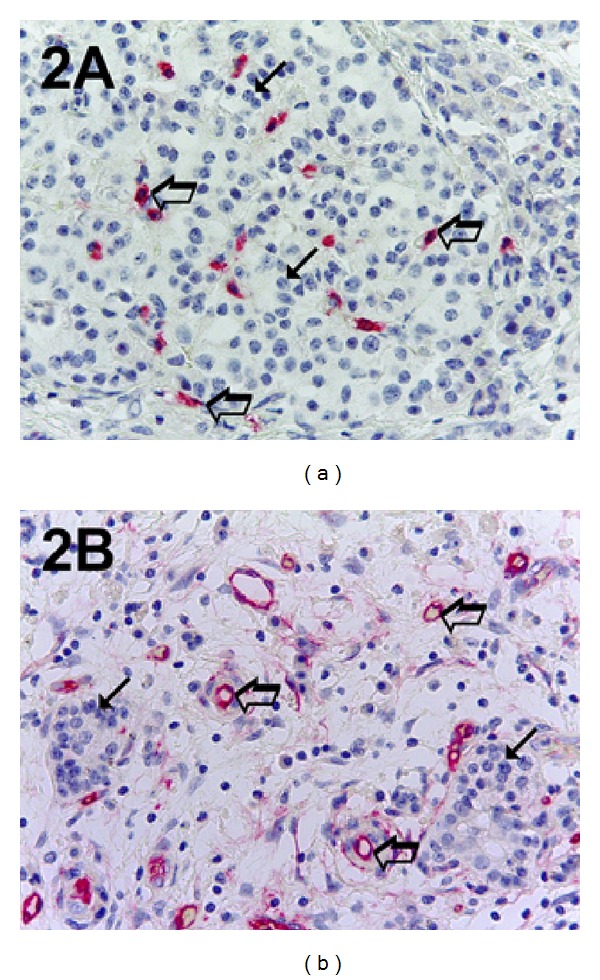
In (a) pancreatic ductal adenocarcinoma sample stained with the anti-tryptase antibody. Many scattered red immunostained MCs. Big arrows indicate single red MC and small arrows indicate the bleu nucleus of cancer cells. In (b) a highly vascularized pancreatic ductal adenocarcinoma sample stained with the anti-CD-31 antibody. Big arrows indicate single red microvessels with a lumen and small arrows indicate the bleu nucleus of cancer cells. Magnification: (a-b), ×400.

**Figure 3:**
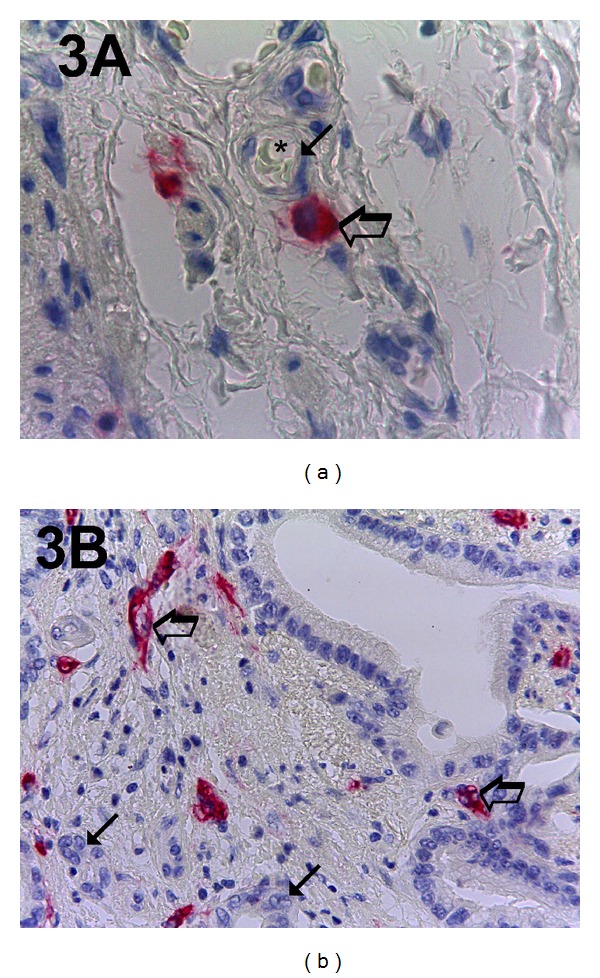
In (a) pancreatic ductal adenocarcinoma sample stained with the anti-tryptase antibody. Big arrow indicates a single red MC and small arrow indicates a microvessel with its lumen. The lumen is marked with an asterisk and there are well visible intraluminal red blood cells. In (b) a highly vascularized pancreatic ductal adenocarcinoma sample stained with the anti-CD-31 antibody. Big arrows indicate single red microvessels with their own lumen and small arrows indicate the bleu nucleus of cancer cells. Magnification: (a-b), ×1000 in oil.

The five most vascularized areas (“hot spots”) were selected at low magnification and both MCDPT (Figure 1(a)) and individual vessel (Figure 1(b)) were counted at ×400 magnification (0.19 mm^2^ area; Figures 2(a) and 2(b)) (GR and NZ) [1]. Single red stained endothelial cells, endothelial cell clusters and microvessels, clearly separated from adjacent microvessels, tumor cells, and other connective tissue elements were counted [17]. Areas of necrosis were not considered for counting. In serial sections each single MC positive to tryptase was counted. Single red stained endothelial cells and red MCs positive to tryptase were also evaluated in terms of immunostained area at ×400 magnification (0.19 mm^2^ area) [17]. Finally morphological detail of both MCs positive to tryptase and endothelial cells was observed at ×1000 magnification in oil (Figures 3(a) and 3(b)).

### 2.4. Statistical Analysis

Linear correlations between MCDPT, MCAPT, MVD, and EA groups were quantified by means of Pearson's correlation coefficient (*r*). Correlation between MCDPT, MCAPT, MVD, and EA groups and the main clinicopathological features were analysed by chi-square test. In all analyses a *P* < 0.05 was considered significant. All statistical analyses were performed with the SPSS statistical software package (SPSS, Inc., Chicago, IL).

## 3. Results

Immunohistochemical staining by using the antibodies anti-CD31 and anti-tryptase allows demonstration of that in highly vascularized cancer tissue; MCs positive to tryptase are well recognizable and generally they are located in perivascular position (Figure 3(a)).

**Figure 4:**
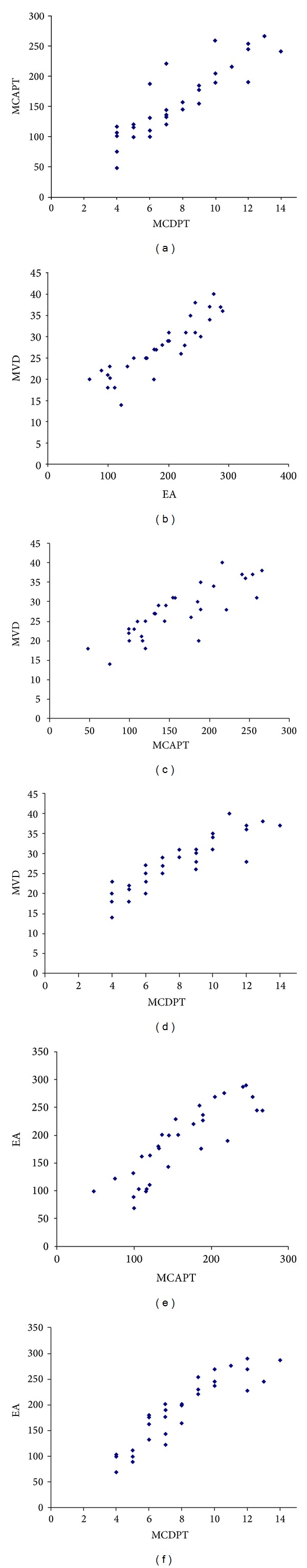
Correlation analysis between MCDPT and MVD (*r* = 0.81; *P* = 0.001), MCAPT and MVD (*r* = 0.69; *P* = 0.003), MCDPT and EA (*r* = 0.76; *P* = 0.002), MCAPT and EA (*r* = 0.73; *P* = 0.002), MVD and EA (*r* = 0.80; *P* = 0.001), and MCDPT and MCAPT (*r* = 0.77; *P* = 0.001).

Mean values ± 1 SD of all the tissue evaluated parameters are reported in Table 2. There was a significant correlation between MCDPT and MVD (*r* = 0.81; *P* = 0.001), between MCAPT and MVD (*r* = 0.69; *P* = 0.003), between MCDPT and EA (*r* = 0.76; *P* = 0.002), between MCAPT and EA (*r* = 0.73; *P* = 0.002), between MVD and EA (*r* = 0.80; *P* = 0.001), and between MCDPT and MCAPT (*r* = 0.77; *P* = 0.001) (Figure 4). No correlation concerning MCDPT, MCAPT, MVD, EA, and the main clinicopathological features was found.

## 4. Discussion

MCs' involvement in tumour angiogenesis has been demonstrated in several animals models and human malignancies [10–14, 18–20].

MCs are recruited and activated via several factors secreted by tumour cells, such as the C-Kit receptor or stem cells factor, VEGF, FGF-2, and TP. In tumour microenvironment, MCs secrete both gelatinases A and B which, in turn, degrade extracellular matrix, releasing stored angiogenic factors [21–33].

On the other hand, MCs may induce angiogenesis by several proangiogenic factors stored in their secretory granules, such as VEGF, FGF-2, tumour necrosis factor alpha, and interleukin 8, transforming growth factor beta, heparin, and tryptase. With special reference to the last, it is involved in tumour angiogenesis stimulating the formation of vascular tubes in* in vitro* and* in vivo* experimental models and it is also an agonist of the PAR-2 in vascular endothelial cells that, in turn, induces angiogenesis. Interestingly in several human malignancies but not in pancreatic cancer, MCDPT and MCAPT have been associated with tumour angiogenesis. In this regard experimental results suggested that MCDPT may stimulate pancreatic cancer cells contributing to pancreatic tumour progression [34–40].

Published data from Esposito et al. [41] showed that mononuclear inflammatory cells of the nonspecific immune response are recruited in pancreatic cancer tissues and they are able to stimulate angiogenesis and cancer progression.

In this pilot study, we have evaluated the correlations between MCDPT, MCAPT, MVD, and EA in a series of 31 PDACP having undergone surgery and our results suggest an association between tryptase and microvascular bed. We found this correlation in double way: first in terms of number of positive tryptase cells and immunostained microvessels and second in terms of extension of positive tryptase area and immunostained microvessels area. To avoid methodological bias the evaluation of MCDPT, MCAPT, MVD, and EA has been performed by means of an image analysis system at ×400 magnifications in a well-defined microscopic area of 0.19 mm^2^ as previously published in other tumours types [1]. Our preliminary data agree on the biological role of tryptase as a strong proangiogenic factor. In this manner we suggest that tryptase from MCs may play a role also in pancreatic tumour tissue angiogenesis. Further studyin a large series of patients will be necessary to confirm our first results. In this context, the evaluation of MCs positive to tryptase may be a novel surrogate angiogenic marker in pancreatic cancer able to predict angiogenic index. We hypothesize also to stop pancreatic angiogenesis inhibiting mast cell degranulation by means of C-Kit inhibitors or targeting tryptase by means of gabexate mesilate or nafamostat mesilate [42–45]. Further studies in more large series of patients are awaited regarding this very intriguing topic.

## Figures and Tables

**Table 1:** Clinicopathological features of patients.

	*N*
Overall series	31
Age	
(i) <65	23
(ii) >65	8
Sex	
(i) Male	25
(ii) Female	6
Tumour site	
(i) Head	13
(ii) Body-Tail	18
TNM by AJCC for Pancreas Cancer Staging 7th Edition	
(i) T_2_N_0-1_M_0_	14
(ii) T_3_N_0-1_M_0_	17
Histologic type	
Ductal adenocarcinomas	31
Histologic grade	
(i) G1-G2	19
(ii) G3	12

**Table 2:** MCAPT, MCDPT, EA, and MVD means ± 1 standard deviations.

MCDPT ×400 magnification (0.19 mm^2^ area)	MCAPT ×400 magnification (0.19 mm^2^ area)	EA ×400 magnification (0.19 mm^2^ area)	MVD ×400 magnification (0.19 mm^2^ area)
8 ± 3^a^	159.38*μ* ^2a^ ± 58.30^a^	186.06*μ* ^2a^ ± 65.89	27 ± 8^a^

^a^Mean ± 1 standard deviation.
